# Multiple ITS Copies Reveal Extensive Hybridization within *Rheum* (Polygonaceae), a Genus That Has Undergone Rapid Radiation

**DOI:** 10.1371/journal.pone.0089769

**Published:** 2014-02-27

**Authors:** Dongshi Wan, Yongshuai Sun, Xu Zhang, Xiaotao Bai, Jun Wang, Ailan Wang, Richard Milne

**Affiliations:** 1 Gansu Key Laboratory of Biomonitoring and Bioremediation for Environmental Pollution, School of Life Sciences , Lanzhou University, Lanzhou, Gansu, P. R. China; 2 School of Life and Sciences, Ludong University, Yantai, Shandong, P. R. China; 3 Institute of Molecular Plant Sciences, School of Biological Sciences, The University of Edinburgh, Edinburgh, United Kingdom; 4 College of Life Science, Sichuan University, Chengdu, Sichuan, P. R. China; Chinese Academy of Medical Sciences, Peking Union Medical College, China

## Abstract

**Background:**

During adaptive radiation events, characters can arise multiple times due to parallel evolution, but transfer of traits through hybridization provides an alternative explanation for the same character appearing in apparently non-sister lineages. The signature of hybridization can be detected in incongruence between phylogenies derived from different markers, or from the presence of two divergent versions of a nuclear marker such as ITS within one individual.

**Methodology/Principal Findings:**

In this study, we cloned and sequenced ITS regions for 30 species of the genus *Rheum*, and compared them with a cpDNA phylogeny. Seven species contained two divergent copies of ITS that resolved in different clades from one another in each case, indicating hybridization events too recent for concerted evolution to have homogenised the ITS sequences. Hybridization was also indicated in at least two further species via incongruence in their position between ITS and cpDNA phylogenies. None of the ITS sequences present in these nine species matched those detected in any other species, which provides tentative evidence against recent introgression as an explanation. *Rheum globulosum*, previously indicated by cpDNA to represent an independent origin of decumbent habit, is indicated by ITS to be part of clade of decumbent species, which acquired cpDNA of another clade via hybridization. However decumbent and glasshouse morphology are confirmed to have arisen three and two times, respectively.

**Conclusions:**

These findings suggested that hybridization among QTP species of *Rheum* has been extensive, and that a role of hybridization in diversification of *Rheum* requires investigation.

## Introduction

Adaptive radiation events are a significant source of new species, ecological diversity and morphological innovation [1]–[4]. Although such events are well known on oceanic islands [3], [5]–[6], they may also occur on continental landmasses if significant climatic and/or geological upheavals have created new ecological niches [4], [7]–[8]. One event implicated in many such radiations is the uplift of the Qinghai–Tibetan Plateau (QTP) [9]–[14].

Hybridization has been suggested to have greatly contributed to the adaptive radiation of many genera that have large numbers of species within a restricted distributional range [5]–[6], [15]–[17], and the genetic signature of hybridization is visible in many such genera [9], [18]–[23]. Reproductive isolation is often incomplete within species groups derived by recent rapid radiation, permitting hybrid formation [24]–[25]. Moreover, hybrid speciation can confer tolerance of new habitats [26]–[27], and may hence contribute to radiation events where many new niches are available to colonise [28]–[32] or even trigger them [24]. However, determining the exact role of hybridization in adaptive radiation events remains challenging.

*Rheum* L. (Polygonaceae) contains ∼60 species, mainly distributed in the QTP and adjacent regions [33]–[35]. This diversity appears to result from two radiation events, the first around 9.9–12.0 million years ago (Mya) and the second around 5 Mya [14], [36]. There is extensive morphological and ecological variation between species [33]–[35], and certain adaptive traits, such as decumbent habit and “glasshouse” morphology involving translucent bracts, have evolved multiple times in parallel [14], [37]–[39]. However, reticulate evolution due to hybridization might give the impression of a character evolving multiple times, when in fact it only evolved once. However, a possible role for hybridization in the diversification of *Rheum* has not yet been investigated. Furthermore, at least one polyploidization event has occurred, because some species are tetraploid [40]–[43].

The nuclear rDNA internal transcribed spacer (ITS) region, is a universal species-specific marker for plants and fungus [44]–[47] and a popular marker for phylogenetic reconstructions [48]–[52] and biogeographic or other evolutionary fields [53]–[60]. Typically several hundred copies exist within plant genomes, which means that the signature of autopolyploidization [52], [61]–[64] or recent hybridization or introgression[63]–[66] can be detected, unless enough generations have passed for concerted evolution to homogenise all ITS copies within the genome [48], [67]. The signature of past hybridization can also be detected by incongruence with a plastid phylogeny, which already exists for *Rheum* [14].

In this study, we extended our previous examination of the diversification history of the genus *Rheum* [10], [14], [37], [39], [68]. We cloned and sequenced ITS sequences for 30 *Rheum* species, representing all seven sections of this genus. We sought evidence of hybridization via (i) multiple ITS copies and (ii) incongruence between ITS and cpDNA, and used this data to evaluate the extent of hybridization during diversification in *Rheum*.

## Materials and Methods

### 2.1 Plant materials

We collected one accession each of 30 species, representing seven sections of *Rheum* (Table 1). Most of the species examined by Sun et al. [14] for cpDNA were included, except for four which could not be obtained: *R. acuminatum, R. delavayi, R. palaestinum* and *R. tataricum*. Most species samples were collected in the QTP, and voucher specimens were deposited in the herbaria of Northwest Plateau Institute of Biology (HNWP), the Chinese Academy of Science, and School of life Sciences, Lanzhou University, China (Table 1). We selected nine species from five other genera of the Polygonaceae, plus *Limonium sinense* from the Plumbaginaceae, to serve as outgroups to root our phylogenetic analyses.

**Table 1:** List of taxa and sources of plant materials analyzed and accessions of ITS sequences in GenBank.

				GenBank accession		
Taxon	Sources/voucher	Chromosome number: 2n(n)/X		ITS version 1 ITS version 2		
***Rheum L*** *.*						
**Sect. I Rheum**						
*R. webbianum* Royle	Cuori, Xizang/Sn31	22/44		KF258680		
*R. hotaoense* C. Y. Cheng et Kao	Ledu, Qinghai/Y99130-1	?		KF258681		KF258682
*R. australe* D. Don	Xizang, Deqing/Liu 1101	22		KF258683		
*R. franzenbachii* Münt	Baotou, neimeng/Liu hb	22		KF258684		KF258685
*R. wittrockii* Lundstr.	Yili, Xinjiang/Y 99059	44		KF258686		
*R. forrestii* Diels	Dali, Yunnan/Liu 2175	?		KF258687		
*R. likiangense* (L.)Sam.	Yushu, Qinghai/Q99147	22		KF258688		KF258689
*R. lhasaense* A. J. Li et P. K. Hsiao	Sangri, Xizang/Liu 1133	?		KF258690		
*R. compactum* L.	Hami, Xinjiang/X99006	44		KF258691		
*R. rhaponticuml* L.	Geneva, Switzerland/GG001	44		KF258692		
*R. altaicum* A. Los.	Aertai, Xinjiang/Liu xj	44		KF258693		
*R. macrophyllum* J. Q. Liu.	Rikaze, Xizang/Liu6265	22/44		KF258694		
**Sect. II Palmata A. Los.**						
*R. officinale* Baill	Nanchuan, Chongqing/991013	44		KF258695		KF258696
*R. palmatum* L	Kangding, Sichuan/Liu 2082	22		KF258697		
*R. tanguticum* Maxim	Gande, Qinghai/Liu 1773	22		KF258698		KF258699
**Sect. III Acuminata C. Y. Cheng et Kao**						
*R. kialense* Franch	Kangding, Sichuan/Liu 2050	?		KF258700		
**Sect. IV Deserticola Maxim**						
*R. sublanceolatum* Cheng et Kao	Chenduo, Qinghai/Liu 847	?	KF258701			
*R. pumilum* Maxim.	Chenduo, Qinghai/Y 99145	44	KF258702		KF258703	
*R. nanum* Siev. ex Pall.	Balikun, Xinjiang/Y 99129-1	22	KF258704			
*R. tibeticum* Maxim. ex Hook.f.	Qushui, Xizang/Liu 1112	?	KF258705			
**Sect. VI Spiciformia A. Los.**						
*R. spiciforme* Royle	Yeduo, Qinghai/Liu 689	22	KF258706			
*R. moocroftianum* Royle	Yeduo, Qinghai/Liu 688	?	KF258707			
*R. przewalskyi* A. Los.	Huzhu, Qinghai/Q99136	?	KF258708			
*R. rhizostachyum* Schrenk	Sunan, Gansu/Liu 1506	?	KF258709			
*R. reticulatum* A. Los.	Maduo, Qinghai/Liu 820	22	KF258710		KF258711	
*R. rhomboideum* A. Los	Yeduo, Qinghai/Liu ly	22	KF258712			
*R. alpinum* J. Q. Liu.	Kangma, Xizang/Liu 6216	?	KF258713			
**Sect. VII Globulosa C. Y. Cheng et Kao**						
*R. globulosum* Gage	Dazi, Xizang/SN221	?	KF258714			
**Sect. VIII Nobilia A. Los.**						
*R. nobile* Hook f. et Thoms	Linzhi, Qinghai/Liu 1206	22	KF258715			
*R. alexandrae* Batal.	Kangding, Sichuan/Liu 2051	22	KF258716			
**OUTGROUPS**.						
*Polygonum viviparum* L.			GQ339919*			
*Polygonum hookeri* Meisn			JN187112.1*			
*Oxyria digyna* (L.) Hill	Wuding, Sichuan/Liu 2087		FJ154474*			
*Oxyria sinensis* Hemsl			KF258717			
*Rumex crispus* L.			AF338221*			
*Calligonum rubicundum* Bge.			JN187107.1*			
*Calligonum arborescens* Litv.			JN187105.1*			
*Atraphaxis spinosa* L.			JN187102.1*			
*Atraphaxis pungens* (Bieb) Jaub. Et Spacht			JN187100.1*			
*Limonium sinense* L.			EU410356*			

*Indicates sequences that were retrieve from GenBank Dateabase (?, not affirmative or reported). Samples were collected in China except *R. rhaponticuml*.

Chromosomal numbers reported by Jaretzky (1928), Chin and Youngken (1947), Hu et al. (2007) and Liu et al. (2010).

### 2.2 DNA extraction, amplification, cloning and sequencing

Total DNA was extracted from silica dried leaves using a modified CTAB method [69]. The primers used for amplification were ITS1 (5′-TCCGTAGGTGAACCTGCGG-3′) and ITS4 (5′-TCCTCCGCTTATTGATATGC-3′) [70]. The amplification program consisted of an initial template denaturation step at 95 for 5 min, followed by 38 cycles at 94 for 20 s, 50 for 30 s, and 72 for 40 s, and extension at 72 for 5 min. We firstly sequenced the amplification products directly. However, most of these sequences can not be identified exactly, especially for seven species, i.e. *R. hotaoense*, *R. officinale*, *R. tanguticum*, *R. pumilum*, *R. likiangense*, *R. franzenbachii* and *R. reticulatum*, mainly because there are a lot of impurity peaks. So for each of the 30 *Rheum* species examined, the amplified fragments were then ligated and transformed into *Escherichia coli* strain DH5α system using a pUC18 vector (Takara Inc.). At least 15 positive clones for each species were selected and sequenced on an ABI Prism automated sequencer with universal primers M13rev and M13uni. In order to reduce false base substitutions resulting from PCR polymerase mismatch, any polymorphism that was observed in only one clone was removed from the analyses. Sequences were edited and aligned with MegAlign and manually adjusted at two positions that had minor length polymorphism. All sequences have been submitted to GenBank (Table S1 in File S1).

### 2.3 Data analyses

The boundaries of the sequenced ITS (including ITS1, 5.8 S, and ITS2) regions were identified in comparison to *Rumex cripus* ITS sequence from GenBank (Accession number: AF338221), and ITS2 boundaries and alignments were confirmed by Hidden Markov Models, following Keller et al. [71]. Clustal W was used for alignment of sequences initially [72], BioEdit v 5.0.6. [73] was used to refine the alignments manually and to determine the lengths and GC contents of ITS1, 5.8 S, and ITS2 separately. The repeats in the ITS sequences were detected with the Tandem Repeats Finder [74]. For each species, sequences for the eight regions of cpDNA examined by Sun et al.[14] were retrieved (Table S1 in File S1). Thus, two sequence matrixes (ITS and cpDNA) were used in further analyses. All indels in the two matrixes were coded as binary states (0 for absence, and 1 for presence) using the GAPCODER program [75].

### 2.4. Phylogeny analyses

Phylogeny analyses were conducted based on the sequences of entire ITS region, ITS1 region and ITS2 region, respectively. We used MrModeltest 2.0 [76] to choose the most appropriate model for each dataset for the ML and Bayesian analyses (the selected model was GTR + I + G for each analysis). Maximum likelihood analyses were performed using PHYML 3.0 with 1000 bootstraps under the GTRIG model [77]; partial parameterization commands used here: –b 1000 –m GTR –v e –f e –t e –a e –o tlr, see the PHYML manual for detail. MrBayes 3.1.2 was used to perform the Bayesian inference analysis to find the optimal tree topology [78]. Four runs were made, each to 10 million generations, saving every 1000th tree. The parameters of the selected model were optimized during searches as recommended, running two independent Markov chain Monte Carlo (MCMC) chains with one cold and three hot chain searches for each dataset with a 50% ‘burn-in’. MCMC convergence was also explored by examining the Potential Scale Reduction Factor convergence diagnostics [79] for all parameters in the model. The posterior probabilities indicating support values for each branch were also estimated.

### 2.5. Genetic distance between species and between ITS versions

Based on the effects of incomplete concerted evolution of ITS sequences, hybridization can be inferred in the ancestry of any individual that contains copies of ITS that are less similar to one another than each is to a sequence present in another species [80]. Seven species contained multiple copies of ITS, i.e. *R. hotaoense*, *R. officinale*, *R. tanguticum*, *R. pumilum*, *R. likiangense*, *R. franzenbachii* and *R. reticulatum*. We used MEGA5 [81] to compute the p-distance between the two ITS versions within each of these species, and that between each version and the most similar sequence detected across all species. This was done using the minimum differences between sequences.

### 2.6. Checking for pseudogenes

Where multiple ITS copies were detected, we checked each copy for two characters that might indicate it to be a pseudogene. The first was the presence of three 5.8 S motifs present which are necessary to give the ITS2 proximal stem connecting 5.8 S to 28 S its correct functional structure, and whose absence hence indicates pseudogenes [82]–[84]. These comprise a one seed plant specific 14-bp motif common to all seed plants [82], and two that are common to all angiosperms [83]–[84]. The second was lower GC content, which also characterises pseudogenes.

## Results

### 3.1 ITS sequence variation

**Figure 1:**
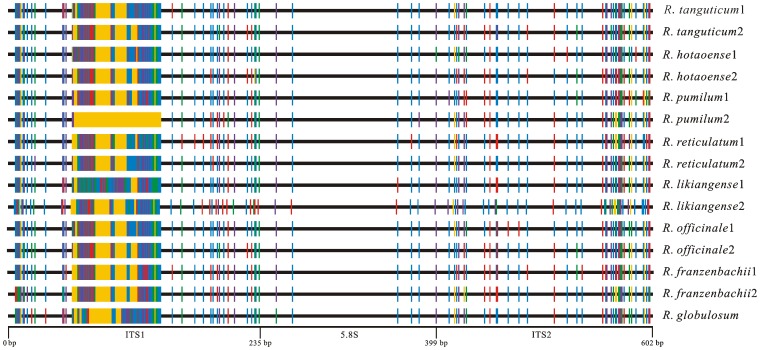
Schematic illustration of the distribution of substitution sites across the entire ITS region obtained from seven species of *Rheum*, using the *R. globulosum* ITS region as reference (red  =  T, purple  =  G, green  =  A, blue  =  C, yellow  =  gap).

The entire ITS region, including ITS1, the 5.8 S rDNA and ITS2, was amplified from at least 15 positive clones per *Rheum* species. Additive polymorphisms were positively identified only when each version of ITS were present in at least two clones per species. Seven accessions contained two highly divergent ITS versions , which differed from one another by at least 27 bases as follows: *R. hotaoense* (33 bases), *R. officinale* (27 bases), *R. tanguticum* (31 bases), *R. pumilum* (71 bases), *R. likiangense* (30 bases), *R. franzenbachii* (27 bases) and *R. reticulatum* (30 bases) (Figure 1). For each of those species, one or two additional accessions were examimed, and every accession was found to contain both versions with different frequency (Table S2 in File S1). In each species, There were two versions of ITS sequences between 2–3 individuals, among sequences of each version, they were different at 2–4 bases, no identical ITS sequences were found, hence there was far greater similarity between individuals than between ITS versions. Furthermore, in each of these species, the two ITS versions were not sister to one another in phylogenetic analysis; for each species, each version for formed a discrete clade not closely related to the other (see below). Single accessions of three other species, i.e *R. nobile*, *R. nanum* and *R. alexandrae*, each contained two barely divergent versions of ITS, that differed at between 1 and 3 sites in each case, and which were strongly supported as reciprocal sister sequences in phylogenetic analysis. In the remaining accessions, only one version was detected. The possibility that any of these ITS sequences could reflect fungal contamination was eliminated following the methods of Li et al. [44].

The ITS sequences of *Rheum* species ranged from 518 bps (*R. pumilum* 2) to 590 bps (*R. nobile*). The ITS1 region ranged from 151 bps (*R. pumilum* 2) to 220 bps (*R. nobile*) (Figure 1). The 5.8 S region had a length of 164 bps in all sequences. The ITS2 region ranged from 202 bps (*R. franzenbachii* 2) to 209 bps (*R. nobile*) annotated according to Keller et al. [71] (Table S3 in File S1). In the seven species, ITS2 regions of two versions between 2–3 individuals of the same species, they were different at 7–16 bases, but with different frquency. Three putative repeats were detected using the ‘Tandem Repeats Finder’ with the default search options (alignment parameters 2, 7, 7, and minimum alignment score 50) (Table 2). These putative repeats were 17–46 bps in length, with 2.1–3.0 copies, and 62–100 percent matches. Among these, two repeats were located in the ITS1 region, and one in the ITS2 (Table 2). The analysis of gene conversion was performed using ‘Gene-conversion Software’ on a basis of the ITS data of all 30 *Rheum* species and no gene conversion was detected.

**Table 2:** Tandem repeats found from ITS sequences of *Rheum*.

Sequence label	Consensus pattern	Consensus size	Copy number	Percent matches	location
*R. rhomboideum*	GACAGACCCGCGAACCCGTCTCTAACCCGCCGTCGGGGCGAGGGGG	46	2.0	100	ITS1
*R. macrophyllum*	AGCGGAGGAAAAGAAAC	17	2.1	100	ITS2
*R. pumilum*	CCCCTCCTGGCGCGCGCCAACCAAA	25	3.2	62	ITS1

To check if any ITS copies might be pseudogenes, for each species with multiple copies, the GC content of each copy was calculated and compared with the *Rumex cripus* ITS region (including ITS1, 5.8 S, and ITS2) , which was designated as the presumed functional paralog. The GC content of ITS of all *Rheum* species possessed similar GC values in the spacers with *Rumex cripus* ITS (Accession number: AF338221, 67.17% in ITS (Table S3 in File S1).

Furthermore, all three of the conserved motifs of 5.8 S, involved in ITS2 proximal stem formation, motif 2 (5′- GAATTGCAGAATCC - 3′), present in all seed plants [82], motif 1 (5′- CGATGAAGAACGTAGC - 3′) and motif 3 (5′- TTTGAACGCA - 3′), both present in all angiosperms [83]–[84] were present and unaltered all investigated sequences (Table S3 in File S1), indicating no loss of function regarding secondary structure formation, and hence that none of the ITS sequences obtained were pseudogenes.

### 3.2 Phylogenetic analyses

When analysed separately, ITS1 and ITS2 each resolved a monophyletic *Rheum* (Figure S1 in File S1), as did a dataset with indels excluded (not shown). Support values were low for most nodes in each analysis, and most differences between the trees therefore might reflect imperfect resolution. Despite this, the very different position of each *R. franzenbachii* version between the two trees is noteworthy, and for this species recombination affecting one ITS version should not be ruled out.

An initial phylogenetic analysis on the combined dataset was conducted using all versions and accessions of ITS for all *Rheum* species (Figure S2 in File S1). However, for those that contained two barely divergent ITS versions, removing one of the two copies had no effect on support values or topology. Likewise, including only one accession per species, for those from which multiple accessions were sampled, had no effect on support values or topology. Therefore, the analysis was re-run using one randomly selected accession per species, and one randomly selected version from those with barely divergent ITS versions, but both versions of those with highly divergent ITS versions.

**Figure 2:**
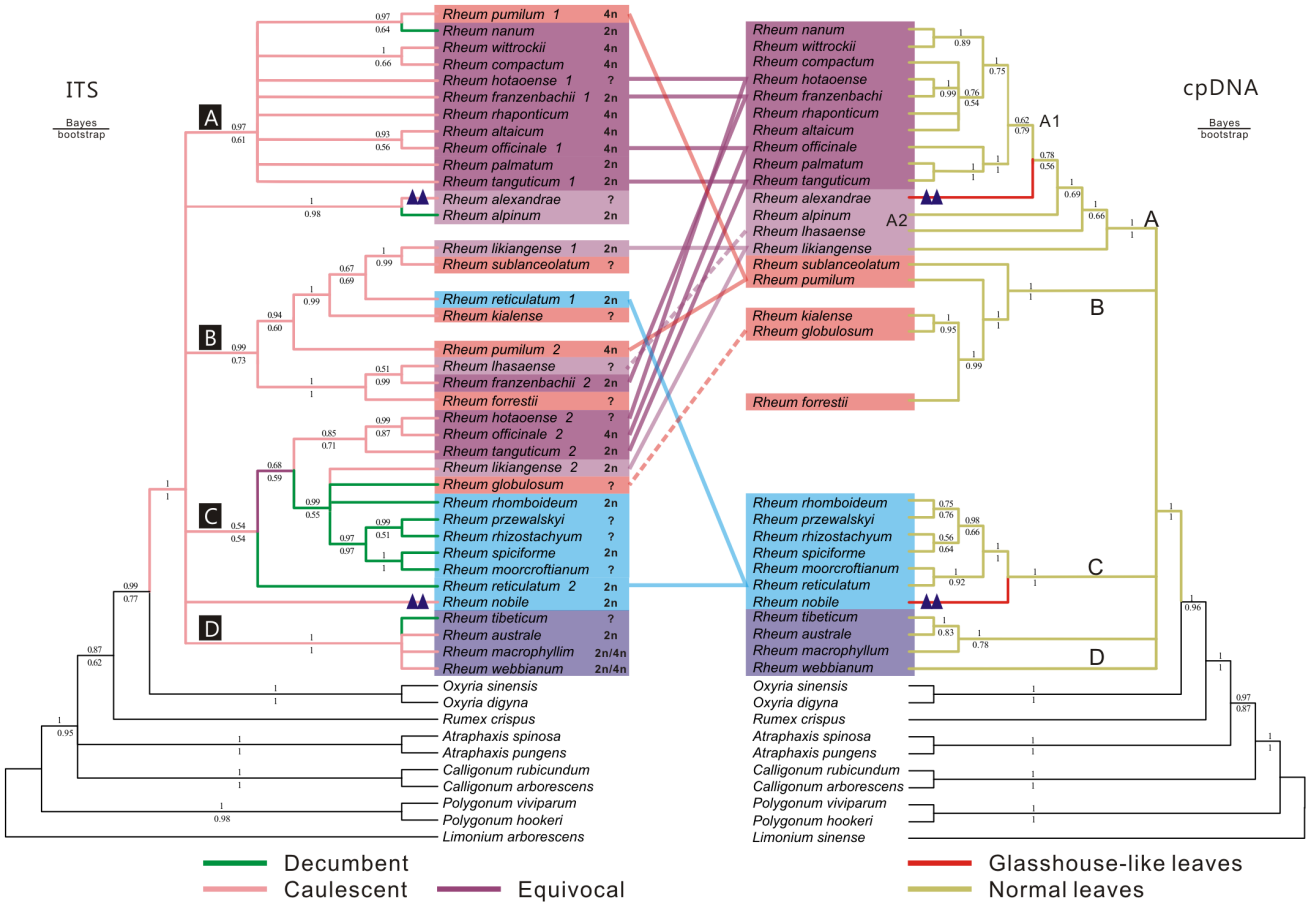
The phylogenetic trees reconstructed using maximum likelihood method on a basis of nrDNA ITS matrix (left, Log-likelihood  = −6967.11) and cpDNA matrix (right, Log-likelihood  = −30277.42). Bootstrap support values from ML analyses using PHYML are given below branches and the corresponding Bayesian posterior probabilities from Bayesian analyses using MrBayes are shown above branches. For simplification, three monophyletic clade A1, A, B, C, D, were marked and also a paraphyletic group A2 on the cpDNA tree. On the ITS tree, four clades and ploidy of each *Rheum* species was marked and the seven species with multiple clones were also marked with clone serial numbers. The different colours branches were used to mark species with different characters, and the branches of glasshouse species was marked with triangle tag.

The Maximum likelihood (ML) trees based on nrDNA ITS and cpDNA are shown in Figure 2, including bootstrap and Bayesian support values. All species from *Rheum* comprised a monophyletic clade sister to the two-species genus *Oxyria* with high support values of 100% (Figure 2). As with cpDNA data [14], four major tentative clades (A, B, C, D) were recovered although the support values for some of them remain low (Figure 2). Within these clades, phylogenetic structure was resolved within B and C, although only a few nodes in each had strong support (Figure 2).

The distinct versions from each of the seven species (*R. hotaoense*, *R. officinale*, *R. tanguticum*, *R. pumilum*, *R. likiangense*, *R. franzenbachii* and *R. reticulatum*) did not cluster together, but nested into different clades with those from other species. Three of them (*R. hotaoense*, *R. officinale*, *R. tanguticum*) had ITS version 1 and cpDNA of Clade A, but ITS version 2 within clade C; *R. franzenbachii* was similar except that ITS version 2 was in Clade B (Figure 2). *R. pumilum* also had ITS version 1 in Clade A, but had both ITS version 2 and cpDNA in Clade B. *R. reticulatum* had ITS version 1 in clade B, but version 2 and cpDNA in Clade C. Most strikingly, *R. likiangense*, was in Clade A for cpDNA, but its two ITS versions were in Clades B and C, respectively (Figure 2).

In addition to these, two species displayed incongruence regarding their clade membership within the two phylogenies. *R. lhasense* was in Clade B for ITS but Clade A for cpDNA, whereas *R. globulosum* was in Clade C for ITS but Clade B for cpDNA. There was also incongruence within Clade C, with for example *R. moorcroftianum* strongly supported as sister to *R. spiciforme* for ITS, but *R. reticulatum* for cpDNA. Incongruence also occurs within Clade B, but is difficult to interpret because clade composition is very different between the two trees (Figure 2).

### 3.3. Pairwise distances

For each of the seven species containing highly divergent versions of ITS, the p-distance between versions was greater than that between each version and the most similar ITS sequence found in another species (Table 3), indicating likely acquisition of a second version via hybridization [80]. Indeed, within-species p-distance was between 19 and 35 for these seven species, whereas the highest between-species p-distance detected was 16 (Table 3).

**Table 3:** p-distances between ITS within and between species, involving species with two divergent versions of ITS.

Species with two versions	Species most similar to version 1	Species most similar to version 2	p-distance:		
			version 1 to version 2	version 1 to nearest sp.	version 2 to nearest sp.
*R. hotaoense*	*R. palmatum*	*R. globulosum*	23	5	15
*R. officinale*	*R. altaicum*	*R. globulosum*	20	6	14
*R. tanguticum*	*R. palmatum*	*R. globulosum*	23	4	11
*R. pumilum*	*R. compactum*	*R. forrestii*	19	15	11
*R. likiangense*	*R. sublanceolatum*	*R. rhoboideum*	35	7	11
*R. franzenbachii*	*R. palmatum*	*R. forrestii*	24	14	11
*R. reticulatum*	*R. sublanceolatum*	*R. globulosum*	26	16	11

## Discussion

ITS data revealed seven instances of *Rheum* species containing two divergent ITS versions, which resolved in different phylogenetic positions in each case (Figure 2), indicating hybridization. Furthermore, incongruence between ITS and a cpDNA phylogeny [14] (Figure 2), regarding the position of *R. lhasaense* and *R. globulosum*, revealed two further instances of hybridization, making nine in total. Other cases of minor incongruence within clades could indicate further hybridization events but other explanations such as lineage sorting effects are possible for these. In each of *R. nobile*, *R. nanum* and *R. alexandrae* , single accessions were found to contain two very similar but not identical versions of ITS; these might be due to ITS variation within species [85] (e.g. divergence between populations followed by gene flow) but do not provide additionally reliable evidence for interspecific hybridization.

### 4.1. Multiple ITS versions in seven *Rheum* species from the QTP

The presence of two ITS versions in seven *Rheum* species (*R. hotaoense*, *R. officinale*, *R. tanguticum*, *R. pumilum*, *R. likiangense*, *R. franzenbachii* and *R. reticulatum*) indicates that in each case a second version has been acquired, and that concerted evolution [86]–[88] has not yet had time to reduce the number of version back to one. This situation has been frequently reported in Angiosperms and is usually attributed to hybridization [52], [61]–[64]. An alternative hypothesis that the extra versions are ITS pseudogenes (see [88]) can be rejected because pseudogenes normally form a distinct clade, having a single common ancestor sequence at the time of duplication, typically before species diversification [89]–[93]; this was not the case for *Rheum*. Furthermore, none of the ITS sequences for the species concerned had lost the conserved seed plant specific 14-bp motif [82] and other two motifs in 5.8 S [83]–[84], or had the lower GC content (Table S1 in File S1), both of which are expected for pseudogenes. Additionally, we sequenced ITS sequences from 2–3 individuals for the seven species to confirm that the multiple copies were not from the sequencing error, and the two versions per species each clustered into two clades with distinct phylogenetic positions (Figure S2 in File S2).

The number of generations necessary for concerted evolution to reach completion is thousands to millions [87], [90], [94]–[97]. The generation time in *Rheum* can be several decades, due to long-lived roots and rhizomes [14], [33]. Therefore, the hybridization events revealed by these multiple ITS versions could be as much as several million years old, and if clonal reproduction makes genets live even longer, they could be older still [52], [61]–[64]. However, recent hybridization is also possible in each case (see below).

All of the seven species with two ITS versions were from the QTP. Among these, two were tetraploid (*R. officinale* and *R. pumilum*), four were diploid, and the chromosome number of *R. hotaoense* is unknown [43]. The tetraploids might be allopolyploids, but could also have received the second ITS copy via introgression following autopolyploidization [28]. Likewise the diploids could be homoploid hybrid species or have acquired a second ITS copy via introgression. Hence in each case three hypotheses are possible: hybrid speciation (allopolyploid or tetraploid depending on the species), ancient introgression, and recent introgression. However, despite this clear evidence for hybridization, the source species for the second ITS versions were not clear. A population genetic strategy based on more samples through its range and the next-generation sequencing method should help us to resolve the hybrid history of the 7 species more clearly.

### 4.2. Other evidence of hybridization in *Rheum*

In addition to these, we found that *R. globulosum* and *R. lhasense* had different phylogenetic positions on the ITS tree to the cpDNA tree (Figure 2). The differing relative positions within Clade B of *R. forrestii* and *R. sublanceolatum* between ITS and cpDNA also suggest possible hybridization for all of these, although the clade composition is so different between the trees that it is difficult to interpret (Figure 2). Small differences between topology within Clade C are also evident, e.g. *R. moocroftianum* has a different sister species in each one. This could reflect lineage sorting in a young clade, or recent hybridization. In particular, the possibility of shared haplotypes between species [98]–[101] needs to be examined in light of this result.

It is noteworthy that neither of the two ITS copies for *R. likiangense* (in clades B and C) has the same phylogenetic position as its cpDNA, which is basal to Clade A. This could reflect more than one hybridization event in its history, and strongly indicates that at least one such event was not recent.

It is likely that not all hybridization events have been detected by this analysis. While concerted evolution towards the paternal ITS type would produce incongruence such as seen here in *R. globulosum* [102], concerted evolution towards the maternal type would remove the signature of hybridization producing congruent phylogenetic positions [48].

### 4.3 Ancient or recent hybridization in *Rheum*?

Ancient introgression is very difficult to distinguish from hybrid speciation, unless large numbers of independent markers are available [103]. This study has uncovered such extensive evidence of hybridization in *Rheum* that a much larger, and more comprehensive data set will be required to unravel it. Of 24 species from the QTP, 9 show unequivocal evidence of hybridization, and there is tentative evidence in many more. There are no cases where pairs or groups of hybridized species are monophyletic for both cpDNA and ITS, therefore there is no evidence in our data for a hybrid speciation event followed by subsequent speciation. However, *R. officinale* and *R. tanguticum* are closely related for both markers, so the possibility cannot entirely be ruled out.

Recent introgression by a single event would lead to all individuals having identical sequences for the captured ITS version, which is not what was observed in any of the species with two divergent ITS versions. Hence if the second versions were acquired by recent introgression then there were multiple, independent introgression events in each affected species. Furthermore, if all instances of multiple ITS versions resulted from recent introgression, then in each case one ITS version would match that for another species, the ITS donor. However, no two ITS sequences detected in this study were identical and the most similar between two species were *R. tanguticum* and *R. palmatum* which differed at 4 loci, which might be due to the limited sample size and the mutation after introgression for ITS loci was unlikely the parsimonious explanation. Although not all members of *Rheum* were examined, those species not examined tended to be narrowly distributed species, and hence unlikely to be recent ITS donors for the accessions with duplicated ITS. Although not unequivocal, this evidence makes it likely that at least some cases of duplicated ITS reflect ancient hybridization events. In particular, the incongruence between both ITS copies and cpDNA for *R. likiangense* cannot easily be explained by recent hybridization.

Morphology also provides clues to past events. Within ITS clade C, all species are decumbent except for four whose second ITS copy falls within this clade. Three of these species (*R. officinale*, *R. hotaoense* and *R. tanguticum*) have both cpDNA and their other ITS copy from Clade A, and also the non-decumbent habit of this clade. From this, introgression of ITS from a Clade C lineage might be a likely hypothesis for their origin. A similar but more complex origin involving a third lineage might apply to *R. likiangense*. Conversely, *R. reticulatum* has ITS from both clades B and C, but contains both cpDNA and the decumbent habit of Clade C, indicating possible introgression of ITS from Clade B. Hence ITS is also consistent with a decumbent habit being ancestral in Clade C, and cpDNA remains congruent with decumbent habit in all five cases above. However, *R. globulosum* has the ITS and decumbent habit of Clade C but the cpDNA of clade B, indicating chloroplast capture as a possible past event for this species. Hence the presence of this species in cpDNA clade B does not indicate an independent origin for the decumbent habit in this clade, as previously thought [14]; instead it is more consistent with a reticulation event. Nonetheless, three separate origins or decumbent habit (Clade C, *R. alpinum* and *R. tibeticum*) are confirmed by the ITS data, and the two separate origins for glasshouse morphology (*R. nobile* and *R. alexandrae*) [14] are also supported.

Minor incongruence within clades, notably Clade C, might reflect more recent and ongoing hybridization events, such as the sharing of plastids between species, which is common among rapidly radiated groups [99]–[100] and other species-rich genera [101]–[102]. Therefore, extensive sampling of different individuals and populations of *Rheum* around the QTP, for both cpDNA and ITS, will be necessary to tease apart the effects of recent and ancient hybridization events. For now we can conclude that hybridization among QTP species of *Rheum* has been extensive, and recent enough for concerted evolution not to have acted in many cases.

## Supporting Information

File S1 Figure S1, The phylogenetic trees reconstructed using maximum likelihood method on a basis of nrDNA ITS1 (left) and ITS2 matrix (right), respectively. Bootstrap support values from ML analyses using PHYML are given below branches and the corresponding Bayesian posterior probabilities from Bayesian analyses using MrBayes are shown above branches. Figure S2, The phylogenetic trees reconstructed using maximum likelihood method on a basis of nrDNA ITS matrix including extra sequences from more individuals. Bootstrap support values from ML analyses using PHYML are given below branches and the corresponding Bayesian posterior probabilities from Bayesian analyses using MrBayes are shown above branches. The letter (X, Y, Z) after the species name present different individuals, and the numbers mean clone order. Table S1, Plant materials and list of accession numbers for the taxa used in the present study. The intron of trnK includes the matK gene and non-coding segments; rbcL-accD and trnL-F are intergenic spacers. Table S2, The frequency of two versions from all positive clones per Rheum species within 2-3 individuals. Table S3, GC content of ITS regions, the character of 5.8 S and ITS2 of Rheum species. Rumex crispus ITS region was used as reference. a present sharing three conserved motifs (motif 1: 5′-CGATGAAGAACGTAGC-3′, motif 2: 5′-GAATTGCAGAATCC-3′ and motif 3: 5′-TTTGAACGCA-3′); b present a stem hybridization; c present homologous structure existed.
(DOC)

## References

[1] Schluter D (2000) Ecological Character Displacement in Adaptive Radiation. Am Nat 156: 4–16.

[2] Gavrilets S, Losos JB (2009) Adaptive radiation: contrasting theory with data. Science 323: 732–737. 19197052 10.1126/science.1157966

[3] Givnish TJ, Millam KC, Theim TT, Mast AR, Theim JT, et al (2009) Origin, adaptive radiation, and diversification of the Hawaiian lobeliads (Asterales: Campanulaceae). Proc Brit Soc B Biol Sci 276: 407–416. 10.1098/rspb.2008.1204 PMC2664350 18854299

[4] Arakaki M, Christin PA, Nyffeler R, Lendel A, Eggli U, et al (2011) Contemporaneous and recent radiations of the world's major succulent plant lineages. Proc Natl Acad Sci USA 108: 8379–8384. 21536881 10.1073/pnas.1100628108 PMC3100969

[5] Grant PR (1986) Ecology and Evolution of Darwin's Finches. Princeton University Press, Princeton, NJ.

[6] Baldwin BG, Sanderson MJ (1998) Age and rate of diversification of the Hawaiian silversword alliance (Compositae). Proc Natl Acad Sci USA 95: 9402–9406. 9689092 10.1073/pnas.95.16.9402 PMC21350

[7] Richardson JE, Pennington RT, Pennington TD, Hollingsworth PM (2001) Rapid diversification of a species-rich genus of neotropical rain forest trees. Science 293: 2242–2245. 11567135 10.1126/science.1061421

[8] Hughes C, Eastwood R (2006) Island radiation on a continental scale: exceptional rates of plant diversification after uplift of the Andes. Proc Natl Acad Sci USA 103: 10334–10339. 16801546 10.1073/pnas.0601928103 PMC1502458

[9] Liu JQ, Wang YJ, Wang AL, Hideaki O, Abbott RJ (2006) Radiation and diversification within the *Ligularia-Cremanthodium-Parasenecio* complex (Asteraceae) triggered by uplift of the Qinghai-Tibetan Plateau. Mol Phylogenet Evol 38: 31–49. 16290033 10.1016/j.ympev.2005.09.010

[10] Wang YJ, Liu JQ, Miehe G (2007) Phylogenetic Origins of the Himalayan Endemic *Dolomiaea*, *Diplazoptilon* and *Xanthopappus* (Asteraceae: Cardueae) Based on Three DNA Regions. Am J Bot 99: 311–322. 10.1093/aob/mcl259 PMC2802998 17218340

[11] Wang LY, Abbott JR, Zheng W, Chen P, Wang YJ, et al (2009) Ltd History and evolution of alpine plants endemic to the Qinghai-Tibetan Plateau: *Aconitum gymnandrum* (Ranunculaceae). Mol Ecol 18: 709–720. 19175501 10.1111/j.1365-294X.2008.04055.x

[12] Mao K, Hao G, Liu J, Adams RP, Milne RI (2010) Diversification and biogeography of *Juniperus* (Cupressaceae): variable diversification rates and multiple intercontinental dispersals. New Phytol 188: 254–272. 20561210 10.1111/j.1469-8137.2010.03351.x

[13] Xu TT, Abbott RJ, Milne RI, Mao KS, Du KF, et al (2010) Phylogeography and allopatric divergence of cypress species (*Cupressus* L.) in the Qinghai-Tibetan Plateau and adjacent regions. BMC Evol Biol 10: 194–203. 20569425 10.1186/1471-2148-10-194 PMC3020627

[14] Sun YS, Wang AL, Wan DS, Wang Q, Liu JQ (2012) Rapid radiation of *Rheum* (Polygonaceae) and parallel evolution of morphological traits. Mol Phylogenet Evol 63: 150–158. 22266181 10.1016/j.ympev.2012.01.002

[15] Sergey G, Losos BJ (2009) Adaptive Radiation: Contrasting Theory with Data. Science 323: 732–737. 19197052 10.1126/science.1157966

[16] Glor ER (2010) Phylogenetic Insights on Adaptive Radiation. Annu Rev Ecol Evol Syst 41: 251–70.

[17] Rowe KC, Aplin KP, Baverstock PR, Moritz C (2011) Recent and rapid speciation with limited morphological disparity in the genus Rattus. Syst Biol 60: 188–203. 21239388 10.1093/sysbio/syq092

[18] Schliewen UK, Klee B (2004) Reticulate sympatric speciation in Cameroonian crater lake cichlids. Front Zool 1: 5. 15679917 10.1186/1742-9994-1-5 PMC544937

[19] Albertson RC, Kocher DT (2005) Genetic architecture sets limits on transgressive segregation in hybrid cichlid fishes. Evolution 59: 686–690. 15856710

[20] Grant PR, Grant BR, Petren K (2005) Hybridization in the Recent Past. Am Nat 166: 56–67. 15937789 10.1086/430331

[21] Masueli RW, Camadro EL, Erazzu LE, Marfil FC (2009) Homoploid hybridization in the evolution of wild diploid potato species. Plant Syst Evol 277: 143–152.

[22] Milne RI, Davies C, Prickett R, Inns LH, Chamberlain FD (2010) Phylogeny of *Rhododendron* subgenus *Hymenanthes* based on Chloroplast DNA markers: between-lineage hybridisation during adaptive radiation? Plant Syst Evol 285: 233–244.

[23] Parsons KJ, Son YH, Albertson RC (2011) Hybridization Promotes Evolvability in African Cichlids: Connections Between Transgressive Segregation and Phenotypic Integration. Evol Biol 38: 306–315.

[24] Seehausen O (2004) Hybridization and adaptive radiation. Trends Ecol Evol 19: 198–207. 16701254 10.1016/j.tree.2004.01.003

[25] Herder F, Nolte WA, Pfaender J, Schwarzer J, Hadiaty KR, et al (2006) Adaptive radiation and hybridization in Wallace's Dreamponds: evidence from sailfin silversides in the Malili Lakes of Sulawesi. Proc R Soc B 273: 2209–2217. 10.1098/rspb.2006.3558 PMC1635519 16901841

[26] Lexer C, Welch ME, Durphy JL, Rieseberg LH (2003) Natural selection for salt tolerance quantitative trait loci (QTLs) in wild sunflower hybrids: Implications for the origin of *Helianthus paradoxus*, a diploid hybrid species. Mol Ecol 12: 1225–1235. 12694286 10.1046/j.1365-294x.2003.01803.x

[27] Ma F, Zhao C, Milne RI, Ji M, Chen LT, et al (2010) Enhanced drought-tolerance in the homoploid hybrid species *Pinus densata*: implication for its habitat divergence from two progenitors. New Phytol 185: 204–216. 19804499 10.1111/j.1469-8137.2009.03037.x

[28] Stebbins GL (1959) The role of hybridization in evolution. Proc Am Phil Soc 103: 231–251.

[29] Rieseberg LH (1995) The Role of Hybridization in Evolution: Old Wine in New Skins. Am J Bot 82: 944–953.

[30] Barton NH (2001) The role of hybridization in evolution. Mol Ecol 10: 551–568. 11298968 10.1046/j.1365-294x.2001.01216.x

[31] Liu JQ, Gao TG, Chen ZD, Lu AM (2002) Molecular phylogeny and biogeography of the Qinghai-Tibet Plateau endemic *Nannoglottis* (Asteraceae). Mol Phylogenet Evol 23: 307–325. 12099790 10.1016/s1055-7903(02)00039-8

[32] Liu JQ (2004) Uniformity of karyotypes in *Ligularia* (Asteraceae: Senecioneae), a highly diversified genus of the eastern Qinghai-Tibet Plateau highlands and adjacent areas. Biol J Linn Soc 144: 329–342.

[33] Losina-Losinskaya AS (1936) The genus *Rheum* and its species. Acta Instituti Botanici Academiae Scientiarum Unionis Rerum Publicarum Soveticarum Socialisticarum, Ser 1: 5–141.

[34] Kao TC, Cheng CY (1975) Synopsis of the Chinese *Rheum* . Acta Phytotax Sin 13: 69–82.

[35] Li AR (1998) Flora Republicae Popularis Sinicae. Science Press, Beijing.

[36] Wang AL, Yang MH, Liu JQ (2005) Molecular Phylogeny, Recent Radiation and Evolution of Gross Morphology of the Rhubarb genus *Rheum* (Polygonaceae) Inferred from Chloroplast DNA *trnL-F* Sequences. Ann Bot 96: 489–498. 15994840 10.1093/aob/mci201 PMC4246783

[37] Zhang DY, Liu BB, Zhao CM, Lu X, Wan DS, et al (2010) Ecological functions and differentially expressed transcripts of translucent bracts in an alpine ‘glasshouse’ plant *Rheum nobile* (Polygonaceae). Planta 231: 1501–1511. 10.1007/s00425-010-1133-x 20221628

[38] Wan DS, Wang AL, Zhang X, Wang ZF, Li ZH (2011) Duplication and adaptive evolution of the *CHS*-like genes of the genus *Rheum* . Biochem Syst Ecol 39: 651–659.

[39] Liu BB, Opgenoorth L, Miehe G, Zhang DY, Wan DS, et al (2013) Molecular bases for parallel evolution of translucent bracts in an alpine “glasshouse” plant *Rheum alexandrae* (Polygonaceae). J Syst Evol 51: 134–141.

[40] Jaretzky R (1928) Histologische und Karyologische Studien on Polygonaceen. Jahrb. Wiss Bot 69: 357–490.

[41] Chin TC, Youngken HW (1947) The Cytotaxonomy of *Rheum.* . Am J Bot 34: 401–407. 20267527

[42] Hu YP, Xie XL (2007) Studies on Karyotypes of Five Populations of *Rheum tanguticum* (Polygonaceae). Acta Bot Yunnanica 29: 429–433.

[43] Liu RR, Wang AL, Tian XM, Wan DS, Liu JQ (2010) Uniformity of katypotypes in *Rheum* (Polygonaceae), a species-rich genus in the Qinghai-Tibetan Plateau and adjacent regions. Caryologia 63: 82–90.

[44] Li DZ, Gao LM, Li HT, Wang H, Ge XJ, et al (2011) Comparative analysis of a large dataset indicates that internal transcribed spacer (ITS) should be incorporated into the core barcode for seed plants. Proc Natl Acad Sci USA 108: 19641–19646. 22100737 10.1073/pnas.1104551108 PMC3241788

[45] Nilsson RH, Ryberg M, Kristiansson E, Abarenkov K, Larsson KH, et al (2006) Taxonomic reliability of DNA sequences in public sequence databases: a fungal perspective. PLoS ONE 1: e59. 17183689 10.1371/journal.pone.0000059 PMC1762357

[46] Schoch CL, Seifert KA, Huhndorf S, Robertd V, Spouge LJ, et al (2012) Nuclear ribosomal internal transcribed spacer (ITS) region as a universal DNA barcode marker for Fungi. Proc Natl Acad Sci USA 109: 6241–6246. 22454494 10.1073/pnas.1117018109 PMC3341068

[47] Ryberg M, Kristiansson E, Sjökvist E, Nilsson RH (2009) An outlook on the fungal internal transcribed spacer sequences in GenBank and the introduction of a web-based tool for the exploration of fungal diversity. New Phytol 181: 471–477. 19121041 10.1111/j.1469-8137.2008.02667.x

[48] Alvarez I, Wendel JF (2003) Ribosomal ITS sequences and plant phylogenetic inference. Mol Phylogenet Evol 29: 417–434. 14615184 10.1016/s1055-7903(03)00208-2

[49] Manen JF (2004) Are both sympatric species Ilex perado and Ilex canariensis secretly hybridizing? Indication from nuclear markers collected in Tenerife. BMC Evol Biol 4: 46. 15550175 10.1186/1471-2148-4-46 PMC535349

[50] Denk T, Grimm GW (2005) Phylogeny and biogeography of Zelkova (Ulmaceae sensu stricto) as inferred from leafmorphology, ITS sequence data and the fossil record. Bot J Linean Soc 147: 129–157.

[51] Besnard G, Rubio de Casas R, Vargas P (2007) Plastid and nuclear DNA polymorphism reveals historical processes of isolation and reticulation in the olive tree complex (*Olea europaea*). J Biogeogr 34: 736–752.

[52] Grimm GW, Denk T (2008) ITS Evolution in *Platanus* (Platanaceae): Homoeologues, Pseudogenes and Ancient Hybridization. Ann Bot 101: 403–419. 18089582 10.1093/aob/mcm305 PMC2701810

[53] Harpke D, Peterson A (2006) Non-concerted ITS evolution in *Mammillaria* (Cactaceae). Mol Phylogenet Evol 41: 579–593. 16843685 10.1016/j.ympev.2006.05.036

[54] Buchheim MA, Keller A, Koetschan C, Förster F, Merget B, et al (2011) Internal transcribed spacer 2 (nu ITS2 rRNA) sequence-structure phylogenetics: towards an automated reconstruction of the green algal tree of life. PLoS ONE 6: e16931. 21347329 10.1371/journal.pone.0016931 PMC3037400

[55] Sang T, Crawford DJ, Stuessy TF (1995) Documentation of reticulate evolution in peonies (*Paeonia*) using internal transcribed spacer sequences of nuclear ribosomal DNA: implications for biogeography and concerted evolution. Proc Natl Acad Sci USA 92: 6813–6817. 7624325 10.1073/pnas.92.15.6813 PMC41419

[56] Porter TM, Golding GB (2011) Are similarity-or phylogeny-based methods more appropriate for classifying internal transcribed spacer (ITS) metagenomic amplicons? New Phytol 192: 775–782. 21806618 10.1111/j.1469-8137.2011.03838.x

[57] Robideau GP, De Cock AW, Coffey MD, Voglmayr H, Brouwer H, et al (2011) DNA barcoding of oomycetes with cytochrome c oxidase subunit I and internal transcribed spacer. Mol Ecol Resour 11: 1002–1011. 21689384 10.1111/j.1755-0998.2011.03041.x PMC3195333

[58] Schultz J, Müller T, Achtziger M, Seibel NP, Dandekar T, et al (2006) The internal transcribed spacer 2 database-a web server for (not only) low level phylogenetic analyses. Nucleic Acids Res 34: W704–W707. 16845103 10.1093/nar/gkl129 PMC1538906

[59] Koetschan C, Hackl T, Müller T, Wolf M, Förster F, et al (2012) ITS2 Database IV: Interactive taxon sampling for internal transcribed spacer 2 based phylogenies. Mol Phylogenet Evol 63: 585–588. 22366368 10.1016/j.ympev.2012.01.026

[60] Poczai P, Hyvönen J (2010) Nuclear ribosomal spacer regions in plant phylogenetics: problems and prospects. Mol Biol Rep 37: 1897–1912. 19626457 10.1007/s11033-009-9630-3

[61] Chen S, Yao H, Han J, Liu C, Song J, et al (2010) Validation of the ITS2 region as a novel DNA barcode for identifying medicinal plant species. PloS ONE 5: e8613. 20062805 10.1371/journal.pone.0008613 PMC2799520

[62] Ochieng JW, Henry RJ, Baverstock PR, Steane DA, Shepherd M (2007) Nuclear ribosomal pseudogenes resolve a corroborated monophyly of the eucalypt genus *Corymbia* despite misleading hypotheses at functional ITS paralogs. Mol Phylogenet Evol 44: 752–764. 17570687 10.1016/j.ympev.2007.04.017

[63] Zheng XY, Cai DY, Yao LH, Teng Y (2008) Non-concerted ITS evolution, early origin and phylogenetic utility of ITS pseudogenes in *Pyrus* . Mol Phylogenet Evol 48: 892–903. 18577457 10.1016/j.ympev.2008.05.039

[64] Xiao LQ, Möller M, Zhu H (2010) High nrDNA ITS polymorphism in the ancient extant seed plant *Cycas*: Incomplete concerted evolution and the origin of pseudogenes. Mol Phylogenet Evol 55: 168–177. 19945537 10.1016/j.ympev.2009.11.020

[65] Griffin AR, Burgess IP, Wolf L (1988) Patterns of natural and manipulated hybridization in the genus *Eucalyptus* L'hérit.-1 a review. Aust J Bot 36: 41–66.

[66] Dumolin-Lape'gue S, Pemonge MH, Gielly L, Taberlet P, Petit RJ (1999) Amplification of DNA from ancient and modern oak wood. Mol Ecol 8: 2137–2140. 10632865 10.1046/j.1365-294x.1999.00788.x

[67] Hardig TM, Brunsfeld SJ, Fritz RS, Morgan M, Orians CM (2000) Morphological and molecular evidence for hybridization and introgression in a willow (Salix) hybrid zone. Mol Ecol 9: 9–24. 10652072 10.1046/j.1365-294x.2000.00757.x

[68] Yang MH, Zhang DM, Zheng JH, Liu JQ (2001) Pollen morphology and its systematic and ecological significance in *Rheum* (the Rhuburb genus, Polygonaceae) from China. Nor J Bot 21: 411–418.

[69] Doyle JJ, Doyle JL (1987) A rapid DNA isolation procedure for small quantities of fresh leaf tissue. Phytochem Bull 19: 11–15.

[70] White TJ, Bruns T, Lee S, Taylor J (1990) Amplification and direct sequencing of fungal ribosomal RNA genes for phylogenetics. In PCR protocols a guide to methods and applications, 315–322. Academic Press, San Diego.

[71] Keller A, Schleicher T, Schultz J, Müller T, Dandekar T, et al (2009) 5.8 S-28 S rRNA interaction and HMM-based ITS2 annotation. Gene 430: 50–57. 19026726 10.1016/j.gene.2008.10.012

[72] Thompson JD, Higgins DG, Gibson TJ (1994) CLUSTAL W: improving the sensitivity of progressive multiple sequence alignment through sequence weighting, position-specific gap penalties and weight matrix choice. Nucleic Acids Res 22: 4673–4680. 7984417 10.1093/nar/22.22.4673 PMC308517

[73] Hall TA (1999) BioEdit, a user-friendly biological sequence alignment editor and analysis program for Windows 95/98/NT. Nucleic. Acids. Symp. Ser 41: 95–98.

[74] Benson G (1999) Tandem repeats finder: a program to analyze DNA sequence. Nucleic Acids Res 27: 573–580. 9862982 10.1093/nar/27.2.573 PMC148217

[75] Young DN, Healy J (2003) GapCoder automates the use of indel characters in phylogenetic analysis. BMC Bioinformatics 4: 1–6. 12689349 10.1186/1471-2105-4-6 PMC153505

[76] Nylander JA (2004) Mr Modeltest v2. Program distributed by the author. Evolutionary Biology Centre, Uppsala University.

[77] Guindon S, Gascuel O (2003) A simple, fast, and accurate algorithm to estimate large phylogenies by maximum likelihood. Syst Biol 52: 696–704. 14530136 10.1080/10635150390235520

[78] Ronquist F, Huelsenbeck JP (2003) MrBayes 3: Bayesian phylogenetic inference under mixed models. Bioinformatics 19: 1572–1574. 12912839 10.1093/bioinformatics/btg180

[79] Gelman A, Rubin DB (1992) Inference from iterative simulation using multiple sequences. Stat Sci 7: 457–472.

[80] Joly S, McLenachan AP, Lockhart JP (2009) A Statistical Approach for Distinguishing Hybridization and Incomplete Lineage Sorting. Am Nat 174: 54–70. 10.1086/600082 19519219

[81] Tamura K, Peterson D, Peterson N, Stecher G, Nei M, et al (2011) MEGA5, Molecular Evolutionary Genetics Analysis Using Maximum Likelihood, Evolutionary Distance, and Maximum Parsimony Methods. Mol Biol Evol 28: 2731–2739. 21546353 10.1093/molbev/msr121 PMC3203626

[82] Jobes DV, Thien LB (1997) A Conserved Motif in the 5.8 S Ribosomal RNA (rRNA) Geneisa Useful Diagnostic Marker for Plant Internal Transcribed Spacer (ITS) Sequences. Plant Mol Biol Rep 15: 326–334.

[83] Harpke D, Peterson A (2007) 5.8 S motifs for the identification of pseudogenic ITS regions. Botany 86: 300–305.

[84] Harpke D, Peterson A (2008) Extensive 5.8 S nrDNA polymorphism in *Mammillaria* (Cactaceae) with special reference to the identification of pseudogenic internal transcribed spacer regions. J Plant Res 121: 261–270. 18373158 10.1007/s10265-008-0156-x

[85] Song J, Shi L, Li D, Sun Y, Niu Y, et al (2012) Extensive pyrosequencing reveals frequent intra-genomic variations of internal transcribed spacer regions of nuclear ribosomal DNA. PLoS ONE 7: e43971. 22952830 10.1371/journal.pone.0043971 PMC3431384

[86] Baldwin BG, Sanderson MJ, Porter JM, Wojciechowski MF, Campbell SC, et al (1995) The ITS region of nuclear ribosomal DNA-A valuable source of evidence on Angiosperm phylogeny. Ann Mo Bot Gard 82: 247–277.

[87] Wolf M, Chen S, Song J, Ankenbrand M, Müller T (2013) Compensatory Base Changes in ITS2 Secondary Structures Correlate with the Biological Species Concept Despite Intragenomic Variability in ITS2 Sequences - A Proof of Concept. PLoS ONE 8: e66726. 23826120 10.1371/journal.pone.0066726 PMC3691174

[88] Koch MA, Dobeš C, Mitchell-Olds T (2003) Multiple Hybrid Formation in Natural Populations: Concerted Evolution of the Internal Transcribed Spacer of Nuclear Ribosomal DNA (ITS) in North American *Arabis divaricarpa* (Brassicaceae). Mol Bio Evol 20: 338–350. 12644554 10.1093/molbev/msg046

[89] Buckler ES, Holtsford TP (1996) Zea systematics: Ribosomal ITS evidence. Mol Biol Evol 13: 612–622. 8882504 10.1093/oxfordjournals.molbev.a025621

[90] Buckler ES, Ippolito A, Holtsford TP (1997) The evolution of ribosomal DNA: Divergent paralogous and phylogenetic implications. Genetics 145: 821–832. 9055091 10.1093/genetics/145.3.821 PMC1207866

[91] Hartmann S, Nason JD, Bhattacharya D (2001) Extensive ribosomal DNA genic variation in the columnar cactus *Lophoce-reus* . J Mol Evol 53: 124–134. 11479683 10.1007/s002390010200

[92] Kita Y, Ito M (2000) Nuclear ribosomal ITS sequences and phylogeny in East Asian *Aconitumsubgenus Aconitum* (Ranunculaceae), with special reference to extensive polymorphism in individual plants. Plant Syst Evol 225: 1–13.

[93] Muir G, Fleming CC, Schlötterer C (2001) Three divergent rDNA clusters predate the species divergence in *Quercus petraea* (Matt.) Liebl. and *Quercus robur* L. Mol Biol Evol 18: 112–119. 10.1093/oxfordjournals.molbev.a003785 11158370

[94] Mayol M, Rosselló JA (2001) Why nuclear ribosomal DNA spacers (ITS) tell different stories in *Quercus* . Mol Phylogenet Evol 19: 167–176. 11341800 10.1006/mpev.2001.0934

[95] Kovarik A, Pires JC, Leitch AR, Lim KY, Sherwood AM, et al (2005) Rapid concerted evolution of nuclear ribosomal DNA in two Tragopogon allopolyploids of recent and recurrent origin. Genetics 169: 931–944. 15654116 10.1534/genetics.104.032839 PMC1449095

[96] Kovarik A, Dadejova M, Lim YK, Chase MW, Clarkson JJ, et al (2008) Evolution of rDNA in *Nicotiana* Allopolyploids: A Potential Link between rDNA Homogenization and Epigenetics. Am J Bot 101: 815–823. 10.1093/aob/mcn019 PMC2710217 18310159

[97] Matyasek R, Tate JA, Lim YK, Srubarova H, Koh J, et al (2007) Concerted evolution of rDNA in recently formed Tropogon allotetraploids is typically associated with an inverse correlation between gene copy number and expression. Genetics 176: 2509–2519. 17603114 10.1534/genetics.107.072751 PMC1950650

[98] Shaw S, Small RL (2005) Chloroplast DNA phylogeny and phylogeography of the North American plums (*Prunus* subgenus *Prunus* section *Prunocerasus*, Rosaceae). Am J Bot 92: 2011–2030. 21646120 10.3732/ajb.92.12.2011

[99] Bänfer G, Moog U, Fiala B, Mohamed M, Weising K, et al (2006) A chloroplast genealogy of myrmecophytic *Macaranga* species (Euphorbiaceae) in Southeast Asia reveals hybridization, vicariance and long-distance dispersal. Mol Ecol 15: 4409–4425. 17107473 10.1111/j.1365-294X.2006.03064.x

[100] McKinnon GE, Steane DA, Potts BM, Vaillancourt RE (1999) Incongruence between chloroplast and species phylogenies in *Eucalyptus* subgenus *Monocalyptus* (Myrtaceae). Am J Bot 86: 1038–1046. 10406727

[101] Kiefer C, Dobes C, Sharbel TF, Koch MA (2009) Phylogeographic structure of the chloroplast DNA gene pool in North American *Boechera*–A genus and continental-wide perspective. Mol Phyl Evol 52: 303–311. 10.1016/j.ympev.2009.03.016 19328239

[102] Lihova J, Shimizu KK, Marhold K (2006) Allopolyploid origin of *Cardamine asarifolia* (Brassicaceae): Incongruence between plastid and nuclear ribosomal DNA sequences solved by a single-copy nuclear gene. Mol Bio Evol 39: 759–786. 10.1016/j.ympev.2006.01.027 16527494

[103] Rieseberg LH, Brunsfeld SJ (1992) Molecular evidence and plant introgression. Pp. 151–178 in P. S. Soltis, D. E. Soltis, and J. J. Doyle, eds. Molecular systematics of plants. Chapman and Hall, New York.

