# Supplementary material for: Multiple ITS Copies Reveal Extensive Hybridization within Rheum (Polygonaceae), a Genus That Has Undergone Rapid Radiation
Source: PLoS One. 2014 Feb 27;9(2):e89769. doi: 10.1371/journal.pone.0089769 (PMC3937351; doi:10.1371/journal.pone.0089769)

Table S1. Plant materials and list of accession numbers for the taxa used in the present study. The intron of *trn*K includes the *mat*K gene and non-coding segments; *rbc*L-*acc*D and *trn*L-F are intergenic spacers.

| Species | GenBank Accession number | | | | | | |
| --- | --- | --- | --- | --- | --- | --- | --- |
|  | *rbc*L | *psa*A | *ndh*F | *acc*D | *rbc*L-*acc*D | *trn*K intron | *trn*L-F |
| *Rheum webbianum* | EU840297 | EU840339 | EU840381 | EU840423 | EU840507 | EU840465 | AY566450* |
| *Rheum forrestii* | EU840304 | EU840346 | EU840388 | EU840430 | EU840514 | EU840472 | AY566447* |
| *Rheum macrophyllum* | EU840308 | EU840350 | EU840392 | EU840434 | EU840518 | EU840476 | EU840546 |
| *Rheum australe* | EU840309 | EU840351 | EU840393 | EU840435 | EU840519 | EU840477 | AY566459* |
| *Rheum lhasaense* | EU840311 | EU840353 | EU840395 | EU840437 | EU840521 | EU840479 | AY566463* |
| *Rheum likiangense* | EU840312 | EU840354 | EU840396 | EU840438 | EU840522 | EU840480 | AF303438* |
| *Rheum franzenbachii* | EU840321 | EU840363 | EU840405 | EU840447 | EU840531 | EU840489 | EU840548 |
| *Rheum hotaoense* | EU840322 | EU840364 | EU840406 | EU840448 | EU840532 | EU840490 | AY566445* |
| *Rheum officinale* | EU840310 | EU840352 | EU840394 | EU840436 | EU840520 | EU840478 | AF303431* |
| *Rheum palmatum* | EU840314 | EU840356 | EU840398 | EU840440 | EU840524 | EU840482 | AY566453* |
| *Rheum tanguticum* | EU840315 | EU840357 | EU840399 | EU840441 | EU840525 | EU840483 | AY566452* |
| *Rheum kialense* | EU840303 | EU840345 | EU840387 | EU840429 | EU840513 | EU840471 | AY566458* |
| *Rheum pumilum* | EU840305 | EU840347 | EU840389 | EU840431 | EU840515 | EU840473 | AF303441* |
| *Rheum tibeticum* | EU840307 | EU840349 | EU840391 | EU840433 | EU840517 | EU840475 | AY566455* |
| *Rheum spiciforme* | EU840293 | EU840335 | EU840377 | EU840419 | EU840503 | EU840461 | AY566461* |
| *Rheum rhomboideum* | EU840294 | EU840336 | EU840378 | EU840420 | EU840504 | EU840462 | EU840544 |
| *Rheum przewalskyi* | EU840295 | EU840337 | EU840379 | EU840421 | EU840505 | EU840463 | AY303442* |
| *Rheum moorcroftianum* | EU840300 | EU840342 | EU840384 | EU840426 | EU840510 | EU840468 | AY566460* |
| *Rheum alpinum* | EU840301 | EU840343 | EU840385 | EU840427 | EU840511 | EU840469 | EU840545 |
| *Rheum globulosum* | EU840302 | EU840344 | EU840386 | EU840428 | EU840512 | EU840470 | AY566449* |
| *Rheum nobile* | EU840298 | EU840340 | EU840382 | EU840424 | EU840508 | EU840466 | AY566465* |
| *Rheum alexandrae* | EU840317 | EU840359 | EU840401 | EU840443 | EU840527 | EU840485 | AY566456* |
| *Rheum sublanceolatum* | EU840306 | EU840348 | EU840390 | EU840432 | EU840516 | EU840474 | AY566454* |
| *Rheum reticulatum* | EU840299 | EU840341 | EU840383 | EU840425 | EU840509 | EU840467 | AY566462* |
| *Rheum rhaponticum* | EU840318 | EU840360 | EU840402 | EU840444 | EU840528 | EU840486 | AY566446* |
| *Rheum rhizostachyum* | EU840296 | EU840338 | EU840380 | EU840422 | EU840506 | EU840464 | AY566448* |
| *Rheum wittrockii* | EU840316 | EU840358 | EU840400 | EU840442 | EU840526 | EU840484 | AY566464* |
| *Rheum compactum* | EU840319 | EU840361 | EU840403 | EU840445 | EU840529 | EU840487 | AF303439* |
| *Rheum altaicum* | EU840320 | EU840362 | EU840404 | EU840446 | EU840530 | EU840488 | EU840547 |
| *Rheum nanum* | EU840313 | EU840355 | EU840397 | EU840439 | EU840523 | EU840481 | AY566444* |
| *Oxyria digyna* | EU840291 | EU840333 | EU840375 | EU840417 | EU840501 | EU840459 | AY566466* |
| *Oxyria sinensis* | JQ342163 | JQ342162 | JQ342161 | JQ342160 | JQ342165 | JQ342164 | JQ342166 |
| *Rumex crispus* | EU840290 | EU840332 | EU840374 | EU840416 | EU840500 | EU840458 | EU840542 |
| *Calligonum rubicundum* | EU840325 | EU840367 | EU840409 | EU840451 | EU840535 | EU840493 | EU840551 |
| *Calligonum arborescens* | EU840326 | EU840368 | EU840410 | EU840452 | EU840536 | EU840494 | EU840552 |
| *Atraphaxis spinosa* | EU840285 | EU840327 | EU840369 | EU840411 | EU840495 | EU840453 | EU840537 |
| *Atraphaxis pungens* | EU840286 | EU840328 | EU840370 | EU840412 | EU840496 | EU840454 | EU840538 |
| *Polygonum hookeri* | EU840289 | EU840331 | EU840373 | EU840415 | EU840499 | EU840457 | EU840541 |
| *Polygonum viviparum* | EU840288 | EU840330 | EU840372 | EU840414 | EU840498 | EU840456 | EU840540 |
| *Limonium sinense* | FJ872106 | FJ872090 | FJ872087 | FJ872083 | FJ872095 | FJ872096 | FJ872102 |

*: These 26 sequences were taken from Wang et al. (2005); all others from Sun et al. (2012).

Table S2 The frequency of two versions from all positive clones per *Rheum* species within 2-3 individuals.

| Species | | *R. pumilum* | | | *R. hotaoense* | | | *R. officinale* | | *R. franzenbachii* | | *R. tanguticum* | | | *R. likiangense* | | | | *R. reticulatum* | | |
| --- | --- | --- | --- | --- | --- | --- | --- | --- | --- | --- | --- | --- | --- | --- | --- | --- | --- | --- | --- | --- | --- |
| Version | | 1 | 2 | 1 | | 2 | 1 | | 2 | 1 | 2 | | 1 | 2 | | 1 | 2 | 1 | | 2 |  |
| Frequency in individuals | X  Y  Z | 10/15  9/15  7/15 | 5/15  6/15  8/15 | 7/15  9/15 | | 8/15  6/15 | 7/15  9/15 | | 8/15  6/15 | 7/15  7/15  9/15 | 8/15  8/15  6/15 | | 6/15  6/15 | 9/15  9/15 | | 7/15  6/15 | 8/15  9/15 | 6/15  9/15  7/15 | | 9/15  6/15  8/15 |  |

Table S3 GC content of ITS regions, the character of 5.8S and ITS2 of *Rheum* species. *Rumex crispus* ITS region was used as reference. a present sharing three conserved motifs (motif 1: 5`-CGATGAAGAACGTAGC-3`, motif 2: 5`-GAATTGCAGAATCC-3` and motif 3 : 5`-TTTGAACGCA-3`); b present a stem hybridization; c present homologous structure existed.

| Clone name | ITS | |  | | 5.8S | | |  | ITS2 | |
| --- | --- | --- | --- | --- | --- | --- | --- | --- | --- | --- |
|  | GC(%) | Length (bps) | |  | Three Motifs^a^ | Proximal stem Structure^b^ | Length (bps) |  | Structure^c^ | Length (bps) |
| *R. pumilum* 1  *R. pumilum* 2 | 66.61  68.42 | 563  513 | |  | +++  +++ | +  + | 164  164 |  | +  + | 206  203 |
| *R. franzenbachii* 1  *R. franzenbachii* 2 | 66.55  66.37 | 556  559 | |  | +++  +++ | +  + | 164  164 |  | +  + | 206  202 |
| *R. hotaoense* 1  *R. hotaoense* 2 | 66.84  68.60 | 561  570 | |  | +++  +++ | +  + | 164  164 |  | +  + | 206  206 |
| *R. officinale* 1  *R. officinale* 2 | 68.66  66.37 | 568  559 | |  | +++  +++ | +  + | 164  164 |  | +  + | 206  206 |
| *R. tanguticum* 1  *R. tanguticum* 2 | 68.84  66.67 | 568  561 | |  | +++  +++ | +  + | 164  164 |  | +  + | 205  206 |
| *R. reticulatum* 1  *R. reticulatum* 2 | 67.97  69.26 | 562  566 | |  | +++  +++ | +  + | 164  164 |  | +  + | 205  204 |
| *R. likiangense* 1 | 68.03 | 563 | |  | +++ | + | 164 |  | + | 204 |
| *R. likiangense* 2 | 68.03 | 588 | |  | +++ | + | 164 |  | + | 206 |
| *R. lhasaense* | 66.55 | 556 | |  | +++ | + | 164 |  | + | 203 |
| *R. alpinum* | 66.49 | 561 | |  | +++ | + | 164 |  | + | 208 |
| *R. tibeticum* | 69.51 | 574 | |  | +++ | + | 164 |  | + | 208 |
| *R. australe* | 69.51 | 574 | |  | +++ | + | 164 |  | + | 208 |
| *R. macrophyllim* | 69.34 | 574 | |  | +++ | + | 164 |  | + | 208 |
| *R. webbianum* | 68.99 | 574 | |  | +++ | + | 164 |  | + | 208 |
| *R. altaicum* | 66.61 | 563 | |  | +++ | + | 164 |  | + | 206 |
| *R. palmatum* | 66.55 | 562 | |  | +++ | + | 164 |  | + | 206 |
| *R. rhaponticum* | 66.90 | 562 | |  | +++ | + | 164 |  | + | 206 |
| *R. compactum* | 66.84 | 564 | |  | +++ | + | 164 |  | + | 206 |
| *R. wittrockii* | 66.43 | 563 | |  | +++ | + | 164 |  | + | 206 |
| *R. forrestii* | 67.09 | 556 | |  | +++ | + | 164 |  | + | 203 |
| *R. nobile* | 69.26 | 566 | |  | +++ | + | 164 |  | + | 209 |
| *R. sublanceolatum* | 68.51 | 562 | |  | +++ | + | 164 |  | + | 204 |
| *R. kialense* | 68.68 | 562 | |  | +++ | + | 164 |  | + | 204 |
| *R. spiciforme* | 67.42 | 574 | |  | +++ | + | 164 |  | + | 205 |
| *R. rhoboideum* | 67.34 | 542 | |  | +++ | + | 164 |  | + | 205 |
| *R. globulosum* | 68.15 | 562 | |  | +++ | + | 164 |  | + | 205 |
| *R. przewalsky* | 67.54 | 573 | |  | +++ | + | 164 |  | + | 205 |
| *R. moorcroftianum* | 67.77 | 574 | |  | +++ | + | 164 |  | + | 205 |
| *R. rhizostachyum* | 66.90 | 568 | |  | +++ | + | 164 |  | + | 205 |
| *R. nanum* | 66.61 | 563 | |  | +++ | + | 164 |  | + | 206 |
| *R. alexandrae* | 66.84 | 561 | |  | +++ | + | 164 |  | + | 208 |
| *Rumex crispus* | 67.17 | 527 | |  | +++ | + | 164 |  | + | 191 |

Figure S1

The phylogenetic trees reconstructed using maximum likelihood method on a basis of nrDNA ITS1 (left) and ITS2 matrix (right), respectively. Bootstrap support values from ML analyses using PHYML are given below branches and the corresponding Bayesian posterior probabilities from Bayesian analyses using MrBayes are shown above branches.

Figure S2

The phylogenetic trees reconstructed using maximum likelihood method on a basis of nrDNA ITS matrix including extra sequences from more individuals. Bootstrap support values from ML analyses using PHYML are given below branches and the corresponding Bayesian posterior probabilities from Bayesian analyses using MrBayes are shown above branches. The letter (X, Y, Z) after the species name present different individuals, and the numbers mean clone order.

Figure S1


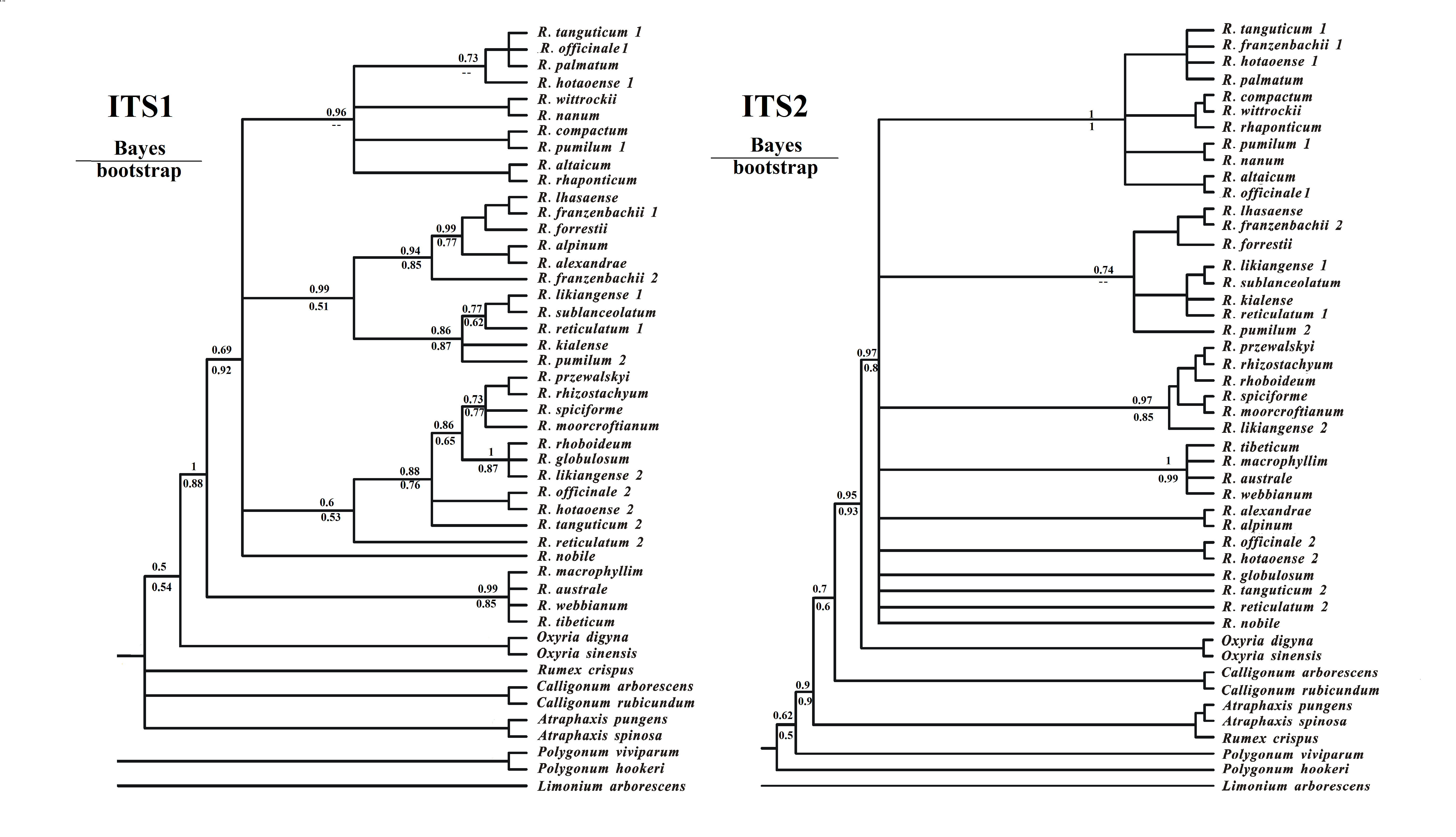


Figure S2


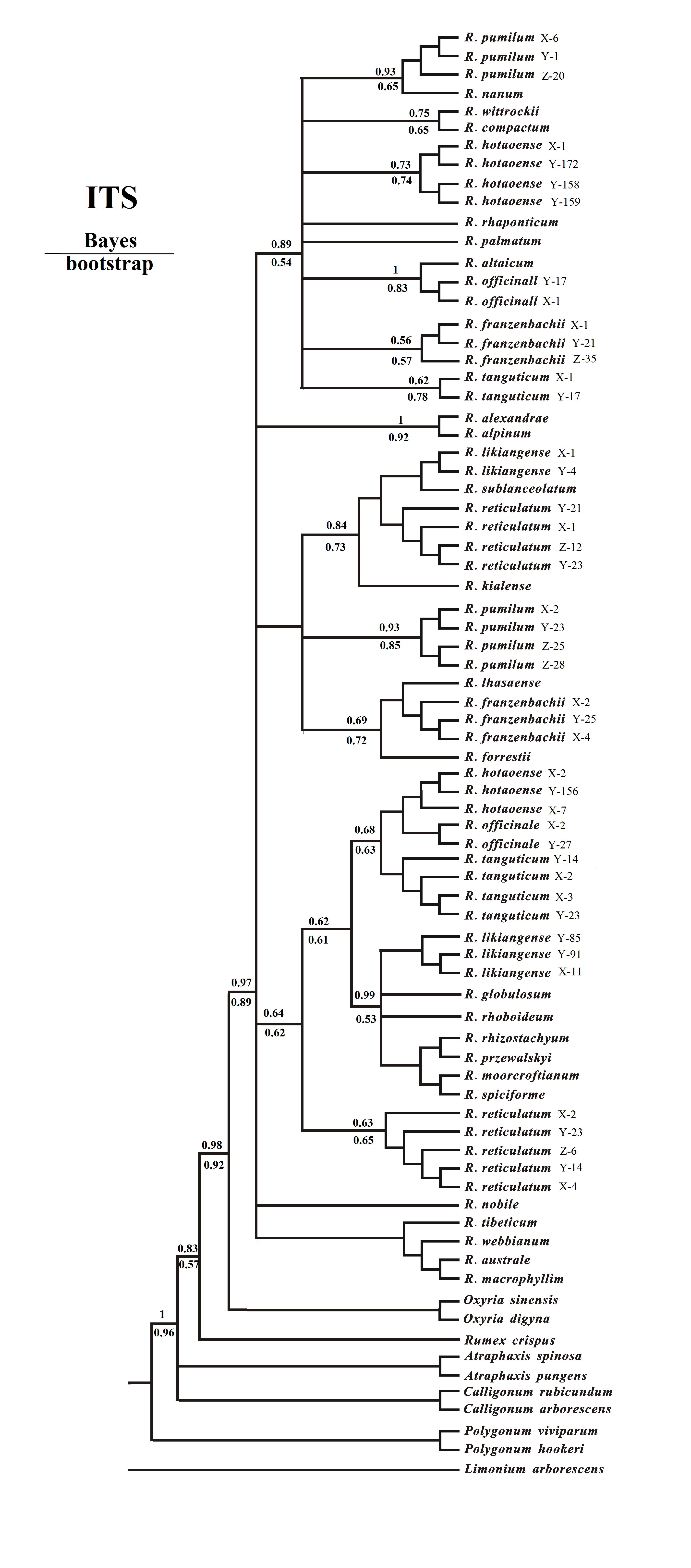

Supplement: File S1 — Figure S1, The phylogenetic trees reconstructed using maximum likelihood method on a basis of nrDNA ITS1 (left) and ITS2 matrix (right), respectively. Bootstrap support values from ML analyses using PHYML are given below branches and the corresponding Bayesian posterior probabilities from Bayesian analyses using MrBayes are shown above branches. Figure S2, The phylogenetic trees reconstructed using maximum likelihood method on a basis of nrDNA ITS matrix including extra sequences from more individuals. Bootstrap support values from ML analyses using PHYML are given below branches and the corresponding Bayesian posterior probabilities from Bayesian analyses using MrBayes are shown above branches. The letter (X, Y, Z) after the species name present different individuals, and the numbers mean clone order. Table S1, Plant materials and list of accession numbers for the taxa used in the present study. The intron of trnK includes the matK gene and non-coding segments; rbcL-accD and trnL-F are intergenic spacers. Table S2, The frequency of two versions from all positive clones per Rheum species within 2-3 individuals. Table S3, GC content of ITS regions, the character of 5.8 S and ITS2 of Rheum species. Rumex crispus ITS region was used as reference. a present sharing three conserved motifs (motif 1: 5′-CGATGAAGAACGTAGC-3′, motif 2: 5′-GAATTGCAGAATCC-3′ and motif 3: 5′-TTTGAACGCA-3′); b present a stem hybridization; c present homologous structure existed. (DOC) [file pone.0089769.s001.doc]
